# Legal demands of the tiotropium bromide for treatment of chronic obstructive pulmonary disease and their financial impact for the State of Paraná, Brazil

**DOI:** 10.31744/einstein_journal/2020GS4442

**Published:** 2019-09-20

**Authors:** Renata Szpak, Giovanna Chipon Strapasson, Beatriz Böger, Yanna Dantas Rattmann, Eliane Carneiro Gomes

**Affiliations:** 1 Universidade Federal do Paraná CuritibaPR Brazil Universidade Federal do Paraná, Curitiba, PR, Brazil.; 2 Centro de Medicamentos do Paraná Secretaria da Saúde do Estado do Paraná CuritibaPR Brazil Centro de Medicamentos do Paraná, Secretaria da Saúde do Estado do Paraná, Curitiba, PR, Brazil.

**Keywords:** Judicial decisions, Pulmonary disease, chronic obstructive, Tiotropium bromide, Unified Health System, Pharmaceutical Services

## Abstract

**Objective:**

To analyze the legal demands of tiotropium bromide to treat chronic obstructive pulmonary disease.

**Methods:**

We included secondary data from the pharmaceutical care management systems made available by the Paraná State Drug Center.

**Results:**

Public interest civil action and ordinary procedures, among others, were the most common used by the patients to obtain the medicine. Two Health Centers in Paraná (Londrina and Umuarama) concentrated more than 50% of the actions. The most common specialty of physicians who prescribed (33.8%) was pulmonology. There is a small financial impact of tiotropium bromide on general costs with medicines of the Paraná State Drug Center. However, a significant individual financial impact was observed because one unit of the medicine represents 38% of the Brazilian minimum wage.

**Conclusion:**

Our study highlights the need of incorporating this medicine in the class of long-acting anticholinergic bronchodilator in the Brazilian public health system.

## INTRODUCTION

Chronic obstructive pulmonary disease (COPD) is a progressive disease that causes limitation to air flow and loss of pulmonary function. This disease is often a result of alveolar abnormality due to significant exposition to particles or toxic gases.^1^

In Brazil, the number of COPD requires updating. However, in 2011, the COPD was the reason of 142,635 hospitalizations and generated R$103 million of expenses to the Brazilian public health system (SUS - *Sistema Único de Saúde* ).^2^ In 2014, COPD was ranked in the third place among the 10 main reasons of death in the world. Smoking was positively correlated to 80% of these deaths.^3^

The recent guidelines of the Global Initiative for Chronic Obstructive Lung Disease (GOLD) proposes a combined review of a number of factors, including drug therapy, smoking cessation, and reduction of pollution and occupational exposition to inhaled irritants.^1 , 4^

The long-acting anticholinergic bronchodilator, such as tiotropium bromide associated with long-acting beta 2-agonist, constitute the preferred drug therapy for different stages of this disease.^5^

In Brazil COPD therapy is part of the Clinical Protocol and Therapeutic Guidelines (CPTG) approved by ordinance nº 609 by the Ministry of Health issued in June 6, 2013. However, no medicine in the class of long-acting anticholinergic bronchodilator, in which tiotropium bromide belongs to, was included in this protocol. For this reason, they are not finding in the National List of Essential Medicines (NLEM) of the SUS,^5 , 6^ and legal action is required to obtain.

The word judicialization has been increasingly used to refer to requests through legal system for the right of access to medicines or other technologies that are not available in the SUS. Historically, these legal demands appeared as mean to obtain the right of access to antiretroviral medicines used by those on treatment for acquired immunodeficiency syndrome (AIDS).^7^

To include, exclude, or change new medicines and technologies at the SUS formulary requires an analysis based on evidence by the National Committee for Health Technology Incorporation (CONITEC) of the SUS. This committee considers, before make a decision, effective aspects such as safety and cost/benefit by comparing medicines, and other technologies that already exist in the system.^8 , 9^

In the State of Paraná, Brazil, the number of patients included due to requested by legal demands, the volume of medicines units distributed, and the public expenses have increased exponentially along the last years. As a consequence, since 2005, the Paraná State Drug Center (CEMEPAR) keeps a department that is responsible for management of medication requests that need to be provided after approval of legal demands.^10^

The guarantee of access to medicines reaffirms the principle of integrality of care in the SUS. In this context, legal demands for medications are fundamental to understand of the SUS, often reflecting the need to incorporate new medications, technologies and updates of clinical protocols by the CONITEC. In this context, the study proposes to investigate actions and cost of legal demands for tiotropium bromide to treat COPD in the State of Paraná, as well as compare results found with clinical protocols of reference of the Ministry of Health^11^ and from GLOBAL.^1^

## OBJETIVE

To investigate the types of actions judged that provide access to tiotropium bromide, and identify what Regional Health Units have the highest number of requests of this medicine, and compare results identified with referral clinical protocol issued by the Ministry of Health and from GLOBAL, and also estimate public and private expenses with this medicine.

## METHODS

This was a retrospective and descriptive exploratory study conducted from 2010 to 2016. Secondary data were obtained using two management systems for pharmaceutical care, the computerized system for management and monitoring of exceptional drugs (SISMEDEX) and the medication management system (SYSMED). Access to this program was possible due to partnership with Paraná State Drug Center (CEMEPAR).

We collected information about number of tiotropium bromide units distributed, type of action, type of medicine demanded by the Regional Health (RH) from State of Paraná, medical specialty that prescribed the medicine, and public and private expenses with the medicine.

Actions considered in the study were: public interest civil action, action for a writ of mandamus, ordinary procedure, and actions classified as “not described” (with no classification in the management system).

Public interest civil action aims to protect collectivity and can be proposed by the Public Ministry by the defense, union, states and municipalities. The action for a writ of mandamus is the action proposed to protect the legal right. Other actions, for example, ordinary procedures, are conduct when the process was not solved by the previous mentioned actions.^12^

The State of Paraná, Brazil, is subdivided in 22 regional health unit (RS): Paranaguá (RS1), Curitiba (RS2), Ponta Grossa (RS3), Irati (RS4), Guarapuava (RS5), União da Vitória (RS6), Pato Branco (RS7), Francisco Beltrão (RS8), Foz do Iguaçu (RS9), Cascavel (RS10), Campo Mourão (RS11), Umuarama, (RS12), Cianorte (RS13), Paranavaí (RS14), Maringá (RS15), Apucarana (RS16), Londrina (RS17), Cornélio Procópio (RS18), Jacarezinho (RS19), Toledo (RS20), Telêmaco Borba (RS21) and Ivaiporã (RS22).

The choice of what medical specialty to include in the study was based on number claims recorded in the Regional Medical Council of Paraná (CRM-PR) related to medical specialty that made the prescriptions for tiotropium bromide.

Public expenses such as medicines and all values in this study are expressed in Brazilian currency (Real).

We included patients diagnosed with COPD and COPD associated to comorbidities, who we were responsible for 96% of demands for the medicine. Those not diagnosed with COPD were excluded from the study.

This study was approved by the Ethical Committee of *Universidade Federal do Paraná,* number 1.812.698, CAAE: 61091416.7.0000.0102.

### Statistical analysis

Data were classified and expressed by descriptive statistics (frequencies and percentages). This statistic analysis was made with the support of statistic package Excel 2016 (Microsoft Excel^®^, EUA), and normality of data was verified using the Kolmogorov-Smirnov tests. Categorical variables were expressed in percentages and compared with χ^2^ test, simulated p value and Fisher’s exact test, as appropriated. P values <5% were considered statistically significant.

For covariant that present significant association, increased by calculus of standard residue of Pearson. Residues higher than 3, in absolute values, indicated independence between variables.

## RESULTS

**Figure 1:**
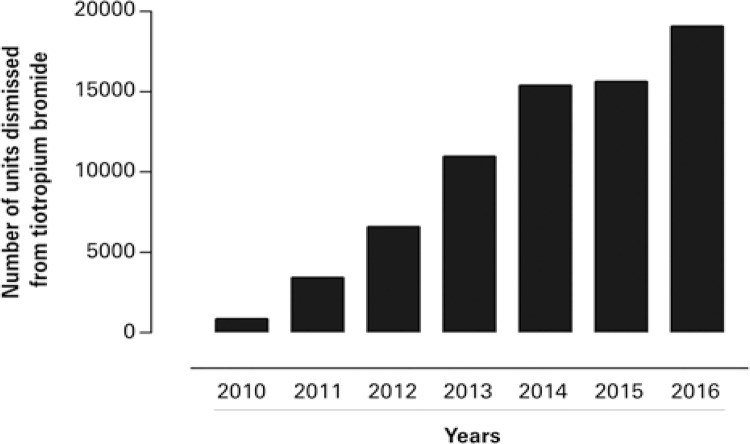
Tiotropium bromide units authorized distribution by legal requests from 2010 to 2016. ICD: International Classification of Diseases and health-related problems

Between 2010 and 2016, the tiotropium bromide units increased more than 61% in number of units distributed in Paraná. In following years, this growth remained between 10 and 24% ( Figure 1 ).

Most frequent actions were public interest civil action (75.3% of total actions), followed by ordinary procedures, among others (23.5% of the total) ( Table 1 ). Action for a writ of mandamus summed less than 4% in all years, and they represented 0.9% of all actions. Not described actions were not only observed until 2012.

**Table 1:** Actions that requested tiotropium bromide in Paraná, Brazil

Type of actions	2010	2011	2012	2013	2014	2015	2016	Total
	n (%)	n (%)	n (%)	n (%)	n (%)	n (%)	n (%)	n (%)
Public interest civil action	129 (88.4)	236 (89.7)	338 (80.9)	364 (71.2)	366 (62.9)	374 (72.8)	343 (81.7)	2.150 (75.3)
Action for a writ of mandamus	1 (0.7)	9 (3.4)	7 (1.7)	4 (0.8)	3 (0.5)	0	1 (0.2)	25 (0.9)
Ordinary procedure	14 (9.6)	16 (6.1)	70 (16.7)	143 (28.0)	213 (36.6)	140 (27.2)	76 (18.1)	672 (23.5)
Not described	2 (1.4)	2 (0.8)	3 (0.7)	0	0	0	0	7 (0.2)

Descriptive statistics.

The State of Paraná, is subdivided in 22RS. Of them, 6 were responsible for approximately 84% of all legal demands for tiotropium bromide in the studied period: RS17 with 36.8%, RS12 with 16.3%, RS2 with 9.5%, RS16 with 9.1%, RS3 with 6.5% and RS15 with 5.7%. Of these, regional of Londrina (RS17) and Umuarama (RS12) drawn the attention because they totalized 53.1% of all medicines obtained through legal demands.

For exploratory analysis, we considered only RS17 and RS12, because they concentrated higher number of actions for medicines demands (n=1,515). We observed a statistic significance (p<0.05) in comparisons between types of predominant actions in RS of Londrina (public interest civil action) and RS of Umuarama (ordinary procedure).

Of the total, 2,854 legal demands generated for tiotropium bromide requested in Paraná, 1,292 (45.3%) belong to a single public interest civil action imposed to Londrina Court, number 2009.70.01.001743-9. It is important to highlight that this action attended municipalities coverage in others RS, according to details observed in table 2 .

**Table 2:** Number of requests included in the public action number 2009.70.01.001743-9 described per Health Region

Health region	Number of coveraged municipalities	Number of demands	Total number of demands	Demands coveraged by the action (%)
Jacarezinho	1	1	464	0.21
Apucarana	1	231	260	88.8
Londrina	19	1.030	1.051	98.0
Cornélio Procópio	15	30	63	47.6

A total of 201 physicians were responsible for prescriptions from 2,854 actions for legal demand for tiotropium bromide in the studied period. Of these physicians, 7 prescribed 1,464 requests (51.2%). Of them, 4 worked in RS in Londrina.

**Figure 2:**
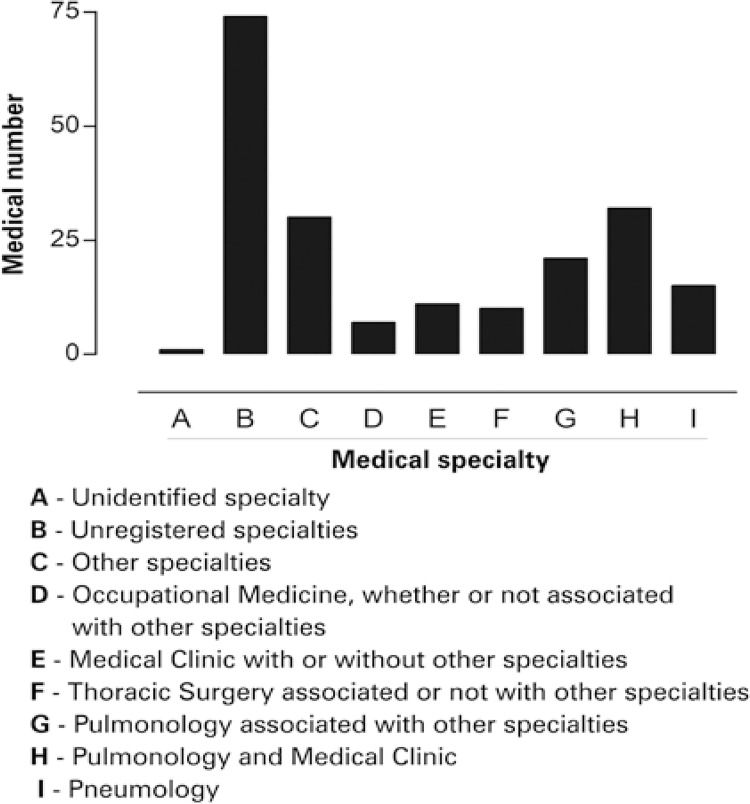
Medical specialty investigated. SESA/PR: Secretary of State of Paraná, Brazil for Health

We observed that 33.8% of physicians who prescribed the medicine were pulmonologists ( Figure 2 ). A total of 36.8% of prescriptions did not have the specialty of the physician who prescribed them.

Expenses with legal demands for tiotropium bromide increased by 97% from 2010 to 2016 ( Table 3 ). The RS of Londrina and RS of Umuarama, together, concentrated the highest expenses.

**Table 3:** Annual expenses, in Brazilian Real, related to legal demands for tiotropium bromide in Regional Health, with emphasis on Londrina (RS17) and Umuarama (RS12)

Regionals of Health	2010	2011	2012	2013	2014	2015	2016
Londrina	45,688.78	249,010.92	526,829.68	755,213.98	858,171.89	943,333.01	706,658,73
Umuarama	32,116.95	98,369.54	126,140.41	195,112.32	305,746.95	368,262.00	513,132.96
Others	33,254.03	119,675.79	274,493.31	704,540.20	1,257.217,64	1,411.237,19	2,190.236,73
Total	111.059.76	467.056.25	927.463.40	1,654.866.50	2,421.136.48	2,722.832.20	3,410.028.42

**Figure 3:**
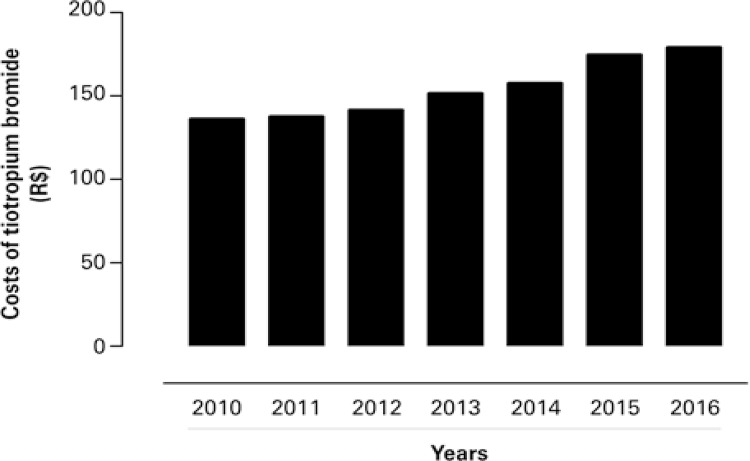
Costs of tiotropium bromide medicine of the Secretary of State of Paraná, Brazil, for Health to attend to legal demands

To respond to legal demands of patients with COPD, the CEMPAR provided monthly a tiotropium bromide unit from Spiriva^®^ Respimat^®^ 2.5mcg/4mL (60 doses). Figure 3 describes the increase of expenses of Secretary of State of Paraná for Health (SESA/PR)^13^ with the medicine.

Costs of medicines determined by Brazil’s National Health Surveillance Agency (ANVISA),^14^ correspond to significant portion of Brazilian minimal wage ( Table 4 ).

**Table 4:** Minimal wage compared with mean cost of medicine

Year	Brazilian minimal wage	Mean cost	%
2010	510	218	42,70
2011	540	243	44,94
2012	622	254	40,88
2013	678	253	37,28
2014	724	264	36,48
2015	788	279	35,42
2016	880	293	33,31
Mean	677	258	38,04

**Figure 4:**
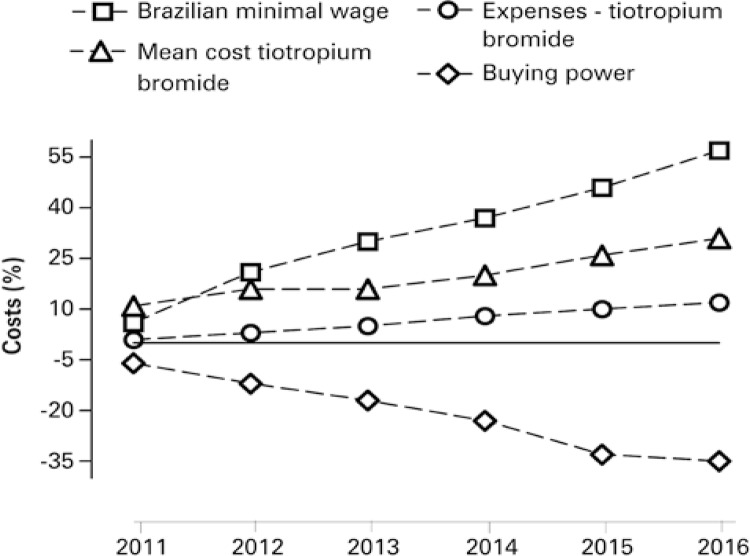
Minimal wage increased and its buying power compared with costs of tiotropium bromide between years 2010 and 2016

Whereas the minimal wage increased by 57% in period considered, the buying power, in the same period, decreased almost 40% ( Figure 4 ).^15^

## DISCUSSION

Tiotropium bromide is medicine with higher number of legal demands in State of Paraná. The absence of this medicine in RENAME provides growing number of actions for treatment of COPD.

In 2013, there was attempt to incorporate tiotropium bromide in RENAME by CONITEC. However, this medicine was not included in the SUS formulary because of possible lack of evidences that represented advantages compared with medicines already available in the SUS formulary.^16^

Studies considered by the CONITEC did not have significant benefits of tiotropium bromide compared with long-acting beta-agonist bronchodilators, or tiotropium bromide associated with corticosteroids. Some studies have presented an increase in the cardiovascular mortality mainly when Respimat^®^ inhalator was used, which is the same made available for administration of tiotropium bromide in Paraná.^16^

In 2013, the proposal for tiotropium bromide incorporation in SUS formulary, estimated a maximal value of sale to the government of R$ 150,29, whereas the mean price of the medicine, according to ANVISA, is R$ 253,00.^14^ For this reason, it is more economically viable to State to include tiotropium bromide in RENAME and made it available to users.^17^

Nunes et al.,^18^ conducted a study about legal demands of medicines in Northeast Region of Brazil. COPD was among the four most prevalent diseases found in actions. The medicine Spiriva^®^ Respimat^®^ was on the list of most requested medicine in actions. These data agree with results obtained in Paraná.

In RS of Umuarana (RS12), predominantly actions were ordinary procedures, which provide more details about the users and their diseases, and also further information such as exams and medical reports.

Public interest civil actions are considered the fastest way to acquire the medicine compared with other types of actions, once they allow benefits for more patients and they can be valid for many years. The majority of demand from our State of Paraná, identified in Regional of Londrina (RS17), was due to a public action from 2009 that provided medicines for 45.3% of legal demands for tiotropium bromide until the end of this study.

Values designed by the State to tiotropium bromide purchase requested by the RS of Londrina and RS of Umuarama are extremely high, especially if compared with total of expenses requested to the purchase of the same medicine in the other 20RS. For example, in 2012, 56.8% of value destined for tiotropium bromide purchase in all cities of State of Paraná was designated to Regional of Londrina (RS17).

During all the period of our study, 50% of tiotropium bromide units made available through legal demand in Paraná were designated to RS of Londrina and RS of Umuarama.

Our investigation related to medical specialties of physicians, who prescribed the medicines, showed a higher predominance of pulmonologists (33.8%). This result corroborates with skills needed to assist most frequent clinical conditions among patients with COPD, *i.e* ., difficult-to-control COPD, considering when patient present two or more episodes of hospitalizations, or admission to emergency units caused by exacerbation in last year; patients who use of inhaled corticosteroids, long-acting beta-agonist or long-acting anticholinergic bronchodilator, those with severe diseases with forced expiratory volume in first second (VEF1) after the use of bronchodilator 30% lower than expected; patients who were symptomatic even using well-established therapy.^19^

Thoracic surgery specialty was described in 5% of actions. This specialty is related to COPD surgically treated such as bullectomy, lung volume reduction and lung transplantation.^20^

Medicines values are often adjusted based on regulation by ANVISA.^14^ The value paid for purchase each unit of tiotropium bromide by SESA-PR increased by 24% in the evaluated period (2010 to 2016). The increase of public expenses with this medicine is considerably lower than cost for personal purchase (by the patient him/herself). In 2016, for example, SESA-PR paid approximately R$ 179,00 per each unit of tiotropium bromide, whereas the patient paid, on average, R$ 293,00 per unit.

Considering the Brazilian minimal wage of each year and the mean cost of tiotropium bromide, we achieved mean values of the medicine for each purchase made by the patient. We can observe that cost per medicine correspond to high proportion of population’s monthly income. For example, in 2011, the tiotropium bromide represented 44.9% of current Brazilian minimal wage. With this result, individual gains with medicine impacted significantly the monthly income of patients. This impact in patients’ income is even higher considering that these medicines are predominantly used by older people, retired individuals, and by those who receive public benefits.

The Brazilian minimal wage increased 57% in the studied period (2010 to 2016), and in the same period the buying power decreased almost 40%. Such situation turns even more difficult the purchase of the medicine by patients.

Considering the ordinance 196, section II of Federal Constitution, attesting that “Health is a right to everyone and obligation of the State”, the growing number of legal demands for tiotropium bromide and their granting are reasonable.^21^

In the States of São Paulo, Minas Gerais, Espirito Santo, Ceará and Distrito Federal, the tiotropium bromide was incorporated in clinical protocols for treatment of COPD. Patients who benefit from this medicine are those at high risk for exacerbation and with persistent symptoms even using long-acting beta-agonist.^16^

Recent international studies confirmed cost-effectiveness of tiotropium bromide administered alone or associated with olodaterol (long-acting beta-agonist). Both cases presented improve in lung function, life quality, reduction of exacerbation and increase of patients’ survival rate.^22 - 24^

From 2010 to 2015, the tiotropium bromide represented 1.7% of total expenses with legal demands assisted by State of Paraná. Although this medicine is the most legally demanded in the State, it is not the medicine with the highest finance impact.

However, considering the private purchase, tiotropium bromide generates a significant finance impact among patients with COPD, the majority of them would not have finance conditions to pay for out-of-pocket treatment, therefore, relaying on the finance support by the State. The debureaucratization allowed by incorporation of tiotropium bromide in the SUS formulary would reduce costs in Paraná and other States, and this would also allow patients to access to a rapid, better, and effective treatment.

## CONCLUSION

The new update to Global Initiative for Chronic Obstructive Lung Disease guidelines, the use of long-acting anticholinergic bronchodilator, such as tiotropium bromide, appears as gold standard treatment for chronic obstructive pulmonary disease.

According to efforts to include the tiotropium bromide in the Brazilian public health system formulary, which was not approved in 2013, we observed that cost of this medicine purchase would be lower than the purchase by the State due to legal demands, which would represent a significant savings in public resources.

In this study, we could observe the need of revision the clinical protocol for chronic obstructive pulmonary disease treatment (in 2013) and of need of include new therapeutic options in National List of Essential Medicines of the Brazilian public health system, such as the inclusion of tiotropium bromide. The inclusion of this medicine is justified because of high number of legal demands, as well as to the finance impact that this medicine cause when purchase by the patient.

## References

[1] Global Initiative for Chronic Obstructive Lung Disease (GOLD). A Guide for health care professionals global initiative for chronic obstructive disease. Pocket guide to copd diagnosis, management, and prevention. A Guide for Health Care Professionals [Internet]. 2017 [cited 2018 Oct 22]. Available from: https://goldcopd.org/wp-content/uploads/2016/12/wms-GOLD-2017-Pocket-Guide.pdf

[2] Rabahi MF. Epidemiologia da DPOC: enfrentando desafios. Pulmão RJ. 2013; 22(2):4-8.

[3] José BP, Corrêa RA, Malta DC, Passos VM, França EB, Teixeira RA, et al. Mortalidade e incapacidade por doenças relacionadas à exposição ao tabaco no Brasil, 1990 a 2015. Rev Bras Epidemiol. 2017;20(20 Suppl 1):75-89. 10.1590/1980-5497201700050007 28658374

[4] Self TH, Ellingson S. New treatment option for chronic obstructive pulmonary disease: two long-acting bronchodilators in a single metered-dose inhaler. Am J Med. 2017;130(11):1251-4. 10.1016/j.amjmed.2017.07.011 28757318

[5] Fernandes FL, Cukier A, Camelier AA, Fritscher CC, Costa CH, Pereira ED, et al. Recomendações para o tratamento farmacológico da DPOC: perguntas e respostas. J Bras Pneumol. 2017;43(4):290-301. 10.1590/S1806-37562017000000153 PMC5687967 29365005

[6] Brasil. Ministério da Saúde. Portaria nº 609, de 6 de junho de 2013. Aprova o Protocolo Clínico e Diretrizes Terapêuticas – Doença Pulmonar Obstrutiva Crônica [Internet]. Diário Oficial da Republica Federativa do Brasil. Brasília (DF); 2013 Jun 7 [citado 2017 Jun 30]. Disponível em: http://portalarquivos.saude.gov.br/images/PDF/2014/junho/13/Portaria-609-de-2013.pdf

[7] Soares JC, Depra AS. Ligações perigosas: indústria farmacêutica, associações de pacientes e as batalhas judiciais por acesso a medicamentos. Physis. 2012;22(1):311-29.

[8] Caetano R, Silva RM, Pedro EM, Oliveira IA, Biz AN, Santana P. Incorporação de novos medicamentos pela Comissão Nacional de Incorporação de Tecnologias do SUS, 2012 a junho de 2016. Cien Saude Colet. 2017;22(8):2513-25. 10.1590/1413-81232017228.02002017 28793068

[9] Santana RS, Lupatini EO, Leite SN. Registro e incorporação de tecnologias no SUS: barreiras de acesso a medicamentos para doenças da pobreza? Cien Saude Colet. 2017;22(5):1417-28. 10.1590/1413-81232017225.32762016 28538914

[10] Conselho Nacional de Secretários de Saúde (CONASS). Para entender a Gestão do SUS - 2015. O enfrentamento das demandas judiciais por medicamentos na secretaria de estado da saúde do Paraná. 1a ed. [Internet]. [citado 2018 Out 22]. Disponível em: http://www.conass.org.br/biblioteca/pdf/colecao2015/CONASS-DIREITO_A_SAUDE-ART_6B.pdf

[11] Brasil. Ministério da Saúde. Protocolos Clínicos e Diretrizes Terapêuticas 2ª edição. Doença Pulmonar Obstrutiva Crônica [Internet]. Secretaria De Atenção À Saúde. Brasília (DF): Ministério da Saúde; 2013 [citado 2018 Mar 15]. Disponível em: http://portalarquivos.saude.gov.br/campanhas/2014/Protocolos_clinicos/publication.pdf

[12] Tribunal de Justiça do Distrito Federal e dos Territórios (TJDFT) [Internet]. Brasília (DF): TJDFT; [citado 2017 Jan 29]. Disponível em: http://www.tjdft.jus.br/

[14] Agência Nacional de Vigilância Sanitária (ANVISA). Consulta Lista de Preço de Medicamento [Internet]. Brasília (DF): ANVISA; 2017 [citado 2017 Jan 30]. Disponível em: http://portal.anvisa.gov.br/listas-de-precos

[15] Instituto Brasileiro de Geografia e Estatística (IBGE). Cálculo da Inflação. Série histórica [Internet]. Rio de Janeiro: IBGE; 2016 [citado 2016 Jul 10]. Disponível em: https://ww2.ibge.gov.br/home/estatistica/indicadores/precos/inpc_ipca/defaultseriesHist.shtm

[16] Brasil. Ministério da Saúde. Secretaria de Ciência, Tecnologia e Insumos Estratégicos. Departamento de Gestão e Incorporação de Tecnologias em Saúde. Brometo de tiotrópio para tratamento da Doença Pulmonar Obstrutiva Crônica. Relatório de Recomendação da Comissão Nacional de Incorporação de Tecnologias no SUS – CONITEC – 68 [Internet]. Brasília (DF): Ministério da Saúde; 2013 [citado 2016 Jul 10]. Disponível em: http://conitec.gov.br/images/Incorporados/BrometoTiotropio-DPOC-final.pdf

[17] Brasil. Portaria nº 533, de 28 de março de 2012. Estabelece o elenco de medicamentos e insumos da Relação Nacional de Medicamentos Essenciais (RENAME) no âmbito do Sistema Único de Saúde (SUS) [Internet]. Diário Oficial da República Federativa do Brasil. Brasília (DF); 2012 Mar 28 [citado 2017 Jun 30]. Disponível em: http://bvsms.saude.gov.br/bvs/saudelegis/gm/2012/prt0533_28_03_2012.html

[18] Nunes CF, Junior AN. Judicialização do direito à saúde na região Nordeste, Brasil: dimensões e desafios. Cad Saude Colet. 2016;24(2):192-9.

[19] Brasil. Ministério da Saúde. Universidade Federal do Rio Grande do SUS - UFRGS. Protocolos de Encaminhamento da Atenção Básica para a Atenção Especializada [Internet]. Brasília (DF): Ministério da Saúde; 2016 [citado 2018 Mar 14]. Disponível em: https://www.ufrgs.br/telessauders/documentos/protocolos_resumos/protocolo_ms_urologia_janeiro_2016.pdf

[20] Franco C, Lear R, Kissmann G. DPOC - o tratamento do paciente grave. Pulmão RJ. 2009;1(1):54.

[21] Brasil. Senado Federal. Constituição (1988). Constituição: República Federativa do Brasil [Internet]. Secretaria de Editoração e Publicações Coordenação de Edições Técnicas. Brasília (DF): Senado Federal; 1988 [citado 2018 Mar 14]. Disponível em: https://www2.senado.leg.br/bdsf/bitstream/handle/id/518231/CF88_Livro_EC91_2016.pdf

[22] Ramadan WH, Kabbara WK, El Khoury GM, Al Assir SA. Combined bronchodilators (tiotropium plus olodaterol) for patients with chronic obstructive pulmonary disease. Int J Chron Obstruct Pulmon Dis. 2015;10(1): 2347-56. 10.2147/COPD.S88246 PMC4634833 26586940

[23] Selya-Hammer C, Gonzalez-Rojas Guix N, Baldwin M, Ternouth A, Miravitlles M, Rutten-van Mölken M, et al. Development of an enhanced health-economic model and cost-effectiveness analysis of tiotropium + olodaterol Respimat^®^fixed-dose combination for chronic obstructive pulmonary disease patients in Italy. Ther Adv Respir Dis. 2016;10(5):391-401. 10.1177/1753465816657272 PMC5933617 27405723

[24] van Boven JF, Kocks JW, Postma MJ. Cost-effectiveness and budget impact of the fixed-dose dual bronchodilator combination tiotropium-olodaterol for patients with COPD in the Netherlands. Int J Chron Obstruct Pulmon Dis. 2016;11:2191-201. 10.2147/COPD.S114738 PMC5036592 27703341

