# Supplementary material for: Legal demands of the tiotropium bromide for treatment of chronic obstructive pulmonary disease and their financial impact for the State of Paraná, Brazil
Source: Einstein (Sao Paulo). 2019 Sep 20;18:eGS4442. doi: 10.31744/einstein_journal/2020GS4442 (PMC6905164; doi:10.31744/einstein_journal/2020GS4442)
Supplement: Translation

# Translation: Demandas judiciais do uso de brometo tiotrópio para o tratamento da doença pulmonar obstrutiva crônica e o impacto financeiro para o Estado do Paraná, Brasil

**Authors:** Renata Szpak, Giovanna Chipon Strapasson, Beatriz Böger, Yanna Dantas Rattmann, Eliane Carneiro Gomes

**Affiliations:** 1 Brasil Universidade Federal do Paraná, Curitiba, PR, Brasil.; 2 Brasil Centro de Medicamentos do Paraná, Secretaria da Saúde do Estado do Paraná, Curitiba, PR, Brasil.

**Keywords:** Decisões judiciais, Doença pulmonar obstrutiva crônica, Brometo de tiotrópio, Sistema Único de Saúde, Assistência farmacêutica

## Abstract

**Objetivo:**

Analisar as demandas judiciais do brometo de tiotrópio para tratar a doença pulmonar obstrutiva crônica.

**Métodos:**

Foram considerados dados secundários dos sistemas gerenciais de assistência farmacêutica, disponibilizados pelo Centro de Medicamentos do Paraná.

**Resultados:**

Ações civis públicas e ações ordinárias, de procedimento comum, entre outras, foram as mais praticadas pelos pacientes para obter o medicamento. Duas Regionais de Saúde do Paraná (Londrina e Umuarama) concentraram mais de 50% das ações. Quanto à especialidade dos médicos prescritores, 33,8% eram pneumologistas. Verificou-se discreto impacto financeiro do brometo de tiotrópio nos gastos gerais com medicamentos pelo Centro de Medicamentos do Paraná. Entretanto, também houve relevante impacto financeiro individual, pois uma unidade do medicamento consome 38% do salário mínimo.

**Conclusão:**

O estudo aponta para a necessidade de incorporação deste medicamento da classe broncodilatadores anticolinérgicos de longa duração, no Sistema Único de Saúde.

## INTRODUÇÃO

A doença pulmonar obstrutiva crônica (DPOC) é caracterizada pela limitação progressiva do fluxo aéreo e pela perda da função pulmonar, geralmente decorrente de anormalidades alveolares causadas pela exposição significativa a partículas ou a gases nocivos”.^1^

No Brasil, os números referentes à DPOC necessitam de atualização. Entretanto, em 2011, a DPOC foi causa de 142.635 internações e gerou gasto de R$103 milhões ao Sistema Único de Saúde (SUS).^2^ No ano de 2014, a DPOC foi classificada em terceiro lugar entre as dez principais causas de morte no mundo. O tabagismo foi positivamente correlacionado a 80% dessas mortes.^3^

As recentes orientações da *Global Initiative for Chronic Obstructive Lung Disease* (GOLD) têm como proposta uma revisão combinada de vários fatores, incluindo a terapia medicamentosa, a cessação do tabagismo, e a redução da poluição e da exposição ocupacional a irritantes inalatórios.^1 , 4^

Em relação à terapia medicamentosa, os broncodilatadores anticolinérgicos de longa ação, como o brometo de tiotrópio, associados aos beta 2 agonista de longa duração, compõem a terapia preferencial em diferentes estágios da doença.^5^

A terapia da DPOC no Brasil está prevista no Protocolo Clínico e Diretrizes Terapêuticas (PCDT) aprovados pela Portaria do Ministério da Saúde 609, de 6 de junho de 2013. Entretanto, nenhum medicamento da classe dos broncodilatadores anticolinérgicos de longa duração, à qual pertence o brometo de tiotrópio, faz parte deste protocolo. Por este motivo, não são encontrados, na Relação Nacional de Medicamentos Essenciais (RENAME) do Sistema Único de Saúde (SUS).^5 , 6^ e recorre-se à judicialização para se obterem estes medicamentos.

A palavra “judicialização” refere-se à crescente procura, de forma judicial, por medicamentos ou outras tecnologias não disponibilizados pelo SUS. As demandas judiciais surgiram historicamente, como meio para obtenção de medicamentos antirretrovirais utilizados no tratamento da síndrome da imunodeficiência adquirida (AIDS).^7^

A incorporação, a exclusão ou a alteração de novos medicamentos e tecnologias no SUS requerem uma análise baseada em evidências pela Comissão Nacional de Incorporação de Tecnologias (CONITEC) no SUS. Esta comissão considera, em suas decisões, aspectos como efetividade, segurança e custo/benefício em comparação aos medicamentos e demais tecnologias já existentes.^8 , 9^

No Estado do Paraná, Brasil, o número de pacientes inseridos nas demandas judiciais, o volume de unidades distribuídas de medicamentos, e os gastos públicos gerados aumentaram exponencialmente nos últimos anos. Como consequência, desde 2005, o Centro de Medicamentos do Paraná (CEMEPAR) mantém um setor exclusivo para gerenciar as solicitações de medicamentos advindos de demandas judiciais.^10^

A garantia de acesso aos medicamentos reafirma o princípio da integralidade do SUS. Neste contexto, as demandas judiciais por medicamentos despertam grande interesse para o entendimento da Saúde Pública no Brasil, refletindo, frequentemente, a necessidade de incorporação de novos medicamentos, tecnologias e atualização de protocolos clínicos pela CONITEC. Neste contexto, o estudo se propôs a investigar as ações e os custos das demandas judiciais do medicamento brometo de tiotrópio para tratar a DPOC no Estado do Paraná, bem como confrontar os resultados encontrados com os protocolos clínicos de referência do Ministério da Saúde^11^ e da GLOBAL.^1^

## OBJETIVO

Realizar um levantamento dos tipos de ações ajuizadas para o medicamento brometo de tiotrópio, investigar as Regionais de Saúde que mais solicitaram o medicamento, confrontar os resultados encontrados com os protocolos clínicos de referência do Ministério da Saúde e da GLOBAL, e inferir os gastos públicos e individuais envolvidos.

## MÉTODOS

Trata-se de um estudo retrospectivo, de caráter exploratório descritivo, que aborda o período de 2010 a 2016. Os dados secundários foram obtidos por meio de dois sistemas gerenciais de assistência farmacêutica: Sistema de Administração de Medicamentos Excepcionais (SISMEDEX) e o Sistema de Administração de Medicamentos (SYSMED). Esse acesso foi viabilizado por meio de parceria com o Centro de Medicamentos do Paraná (CEMEPAR).

Foram obtidas informações sobre número de unidades dispensadas do brometo de tiotrópio, tipo de ação, demandas do medicamento por Regionais de Saúde (RS) do Estado do Paraná, especialidade médica dos prescritores, e gastos públicos e individuais com o medicamento.

As ações consideradas no estudo foram dos tipos: ações civis públicas, mandado de segurança, ações de procedimento comum e ações designadas como “não descritas” (não tipificadas no sistema gerencial).

A ação civil pública visa a proteger a coletividade e pode ser proposta pelo Ministério Público, pela defensoria, pela união, pelos Estados e pelos municípios. Mandado de segurança é a ação proposta para proteger o direito líquido e certo. As demais ações, a exemplo das ações de procedimento comum, são realizadas quando o processo não puder ser gerido pelas ações anteriormente citadas.^12^

O Estado do Paraná, Brasil, é subdividido em 22 RS: Paranaguá (RS1), Curitiba (RS2), Ponta Grossa (RS3), Irati (RS4), Guarapuava (RS5), União da Vitória (RS6), Pato Branco (RS7), Francisco Beltrão (RS8), Foz do Iguaçu (RS9), Cascavel (RS10), Campo Mourão (RS11), Umuarama, (RS12), Cianorte (RS13), Paranavaí (RS14), Maringá (RS15), Apucarana (RS16), Londrina (RS17), Cornélio Procópio (RS18), Jacarezinho (RS19), Toledo (RS20), Telêmaco Borba (RS21) e Ivaiporã (RS22).

As especialidades médicas foram investigadas a partir do número de inscrição no Conselho Regional de Medicina (CRM-PR) do prescritor.

Nos gastos públicos com o medicamento, todos os valores foram expressos na moeda brasileira (Reais).

Foram incluídos no estudo pacientes com diagnóstico de DPOC e DPOC associada a comorbidades, os quais foram responsáveis por 96% das demandas. Foram excluídos do estudo os indivíduos que não tinham diagnóstico de DPOC.

Este trabalho possui aprovação do Comitê de Ética da Universidade Federal do Paraná, sob o número do parecer 1.812.698, CAAE: 61091416.7.0000.0102.

### Análise estatística

Os dados foram tabulados e expressos por estatística descritiva (frequências e percentuais). A análise estatística foi feita com o auxílio do pacote estatístico Excel 2016 (Microsoft Excel^®^, EUA), e a normalidade dos dados foi verificada com os testes de Kolmogorov-Smirnov. As variáveis categóricas foram expressas em percentagens e comparadas com o teste do χ^2^ , teste do χ^2^ com valor de p simulado e teste exato de Fisher, conforme apropriado. Valores de p<5% foram considerados estatisticamente significativos.

Para as covariáveis que apresentaram associação significativa, prosseguiu-se com o cálculo do resíduo padronizado de Pearson. Resíduos superiores a três, em valor absoluto, indicam independência entre as variáveis.

## RESULTADOS

Entre 2010 e 2016, o brometo de tiotrópio apresentou aumento de mais de 61% no número de unidades dispensadas no Paraná. Nos anos seguintes, manteve-se com crescimento entre 10 e 24% ( Figura 1 ).

**Figura 1:** Unidades distribuídas do brometo de tiotrópio demandados judicialmente entre os anos de 2010 e 2016. CID: Classificação Internacional de Doenças e Problemas Relacionados à Saúde

As ações mais frequentes foram as civis públicas (75,3% dos processos totais), seguidas de ações de procedimento comum entre outras (23,5% do total) ( Tabela 1 ). Os mandatos de segurança somaram menos de 4% em todos os anos e representaram 0,9% do total de ações. Ações não descritas só foram observadas até o ano de 2012.

**Tabela 1:** Ações que solicitaram o brometo de tiotrópio no Paraná, Brasil

Tipo da ação	2010	2011	2012	2013	2014	2015	2016	Total
	n (%)	n (%)	n (%)	n (%)	n (%)	n (%)	n (%)	n (%)
Ação civil pública	129 (88,4)	236 (89,7)	338 (80,9)	364 (71,2)	366 (62,9)	374 (72,8)	343 (81,7)	2.150 (75,3)
Mandado de segurança	1 (0,7)	9 (3,4)	7 (1,7)	4 (0,8)	3 (0,5)	0	1 (0,2)	25 (0,9)
Ações de procedimento comum	14 (9,6)	16 (6,1)	70 (16,7)	143 (28,0)	213 (36,6)	140 (27,2)	76 (18,1)	672 (23,5)
Não descritas	2 (1,4)	2 (0,8)	3 (0,7)	0	0	0	0	7 (0,2)

O Estado do Paraná, é subdividido em 22 RS. Seis delas foram as responsáveis por aproximadamente 84% das demandas judiciais do brometo de tiotrópio no período abordado: RS17 com 36,8%, RS12 com 16,3%, RS2 com 9,5%, RS16 com 9,1%, RS3 com 6,5% e RS15 com 5,7%. Destas, as regionais de Londrina (RS17) e Umuarama (RS12) destacam-se por totalizarem 53,1% dos processos de demandas judiciais para obtenção do medicamento.

Para análise exploratória, foram consideradas apenas as RS17 e RS12, pois elas concentraram o maior número de processos de demanda do medicamento (n=1.515). Observou-se significância estatística (p<0,05) nas comparações entre os tipos de ações predominantes nas regionais de Londrina e Umuarama, os quais foram, respectivamente, as ações civis públicas e as de procedimento comum.

Do total de 2.854 demandas judiciais geradas para solicitação do brometo de tiotrópio no Paraná, 1.292 (45,3%) pertencem a uma única ação civil pública ajuizada na Vara Federal de Londrina, identificada pelo número 2009.70.01.001743-9. Cabe ressaltar que esta ação atendeu a municípios abrangidos em outras RS, conforme detalhado na tabela 2 .

**Tabela 2:** Quantidade de demandas compreendidas na ação pública número 2009.70.01.001743-9 por Regional de Saúde

Regional de Saúde	Número de municípios abrangidos	Número de demandas	Número total de demandas	Demandas abrangidas pela ação (%)
Jacarezinho	1	1	464	0,21
Apucarana	1	231	260	88,8
Londrina	19	1.030	1.051	98,0
Cornélio Procópio	15	30	63	47,6

No total, 201 médicos foram responsáveis pelas prescrições que fundamentaram os 2.854 processos de demanda judicial do brometo de tiotrópio ao longo do período estudado. Deste total, sete médicos foram os prescritores em 1.464 demandas (51,2%). Quatro destes atuavam na RS de Londrina.

Verificou-se que 33,8% dos médicos prescritores possuem especialidade em pneumologia ( Figura 2 ). Não havia registro da especialidade médica para 36,8% dos prescritores.

**Figura 2:** Especialidades médicas levantadas. SESA/PR: Secretaria de Estado da Saúde do Paraná, Brasil

Os gastos com as demandas judiciais do brometo de tiotrópio aumentaram 97% de 2010 a 2016 ( Tabela 3 ). As RS de Londrina e Umuarama concentram, isoladamente, os maiores gastos.

**Tabela 3:** Gastos anuais, em Reais, com demanda do brometo de tiotrópio nas Regionais de Saúde, com destaque para Londrina (RS17) e Umuarama (RS12)

Regionais de Saúde	2010	2011	2012	2013	2014	2015	2016
Londrina	45.688,78	249.010,92	526.829,68	755.213,98	858.171,89	943.333,01	706.658,73
Umuarama	32.116,95	98.369,54	126.140,41	195.112,32	305.746,95	368.262,00	513.132,96
As demais	33.254,03	119.675,79	274.493,31	704.540,20	1.257.217,64	1.411.237,19	2.190.236,73
Total	111.059,76	467.056,25	927.463,40	1.654.866,50	2.421.136,48	2.722.832,20	3.410.028,42

Em resposta às demandas judiciais de pacientes com DPOC, o CEMEPAR fornecia mensalmente uma unidade do brometo de tiotrópio da marca Spiriva^®^Respimat^®^2,5mcg/4mL (60 doses). A figura 3 representa a evolução dos gastos da Secretaria Estadual de Saúde do Paraná (SESA/PR)^13^ com este medicamento.

**Figura 3:** Valor unitário gasto com o medicamento brometo de tiotrópio pela Secretaria Estadual de Saúde do Paraná, Brasil, em atendimento às demandas judiciais

Os custos do medicamento, determinados pela Agência Nacional de Vigilância Sanitária (ANVISA),^14^ correspondem a uma fração significativa do salário mínimo ( Tabela 4 ).

**Tabela 4:** Salário mínimo em comparação ao custo médio do medicamento

Ano	Salário mínimo	Custo médio	%
2010	510	218	42,70
2011	540	243	44,94
2012	622	254	40,88
2013	678	253	37,28
2014	724	264	36,48
2015	788	279	35,42
2016	880	293	33,31
Média	677	258	38,04

Enquanto o salário mínimo aumentou 57% no período considerado, o poder de compra, no mesmo período, decresceu quase 40% ( Figura 4 ).^15^

**Figura 4:** Evolução do salário mínimo e seu poder de compra comparados aos custos do brometo de tiotrópio entre os anos 2010 e 2016

## DISCUSSÃO

O brometo de tiotrópio é o medicamento com maior número de demandas judiciais no Estado do Paraná. A ausência deste medicamento na RENAME fomenta o crescente número de ações para o tratamento da DPOC.

Em 2013, houve tentativa de incorporação do brometo de tiotrópio na RENAME pela CONITEC. Porém, o medicamento não foi inserido na lista do SUS, por suposta falta de evidências de que representava vantagens em relação aos medicamentos já disponibilizados do SUS.^16^

Os estudos considerados pela CONITEC não apresentaram benefícios significativos do medicamento quando comparados a broncodilatadores beta-agonistas de longa duração, ou estes associados aos corticosteroides. Alguns estudos apresentam o aumento no risco de mortalidade cardiovascular, principalmente quando utilizado com o inalador Respimat^®^, que é mesmo disponibilizado para a administração do medicamento no Paraná.^16^

Na proposta de incorporação no ano de 2013, o valor máximo de venda para o governo seria de R$ 150,29, enquanto o preço médio, conforme ANVISA, seria R$ 253,00.^14^ Portanto, é mais viável economicamente para o Estado inserir o medicamento na RENAME e disponibilizá-lo aos usuários.^17^

Nunes et al.,^18^ realizaram estudo sobre demandas judiciais de medicamentos na Região Nordeste do Brasil. Entre as quatro doenças mais prevalentes nos processos, estava a DPOC. O medicamento Spiriva^®^Respimat^®^constava entre os mais solicitados nas ações. Estes dados corroboram os resultados obtidos no Paraná.

Na RS de Umuarama (RS12), predominaram as ações de procedimento comum, as quais fornecem mais detalhes referentes aos usuários e suas patologias, além de exames e relatórios médicos.

As ações civis públicas são consideradas uma via de acesso mais rápida, quando comparadas aos demais tipos de ação, uma vez que permitem beneficiar mais do que um paciente e podem se manter válidas por muitos anos. A maior demanda do Estado, identificada na regional de Londrina (RS17), decorre de ação civil pública, que data do ano de 2009, e que beneficiou 45,3% dos processos de demanda judicial do brometo de tiotrópio até o momento deste estudo.

Os valores destinados pelo Estado à compra do brometo de tiotrópio demandado pelas RS de Londrina e Umuarama são muito expressivos, sobretudo quando comparados ao total de gastos com o mesmo medicamento nas outras 20RS. Como exemplo, no ano de 2012, 56,8% do valor gasto com o brometo de tiotrópio em todo o Paraná, foi destinado à regional de Londrina (RS17).

Proporcionalmente, em todo o período considerado no estudo, 50% dos frascos dispensados do brometo de tiotrópio, via demanda judicial no Paraná, foram destinados às RS de Londrina e Umuarama.

Este estudo ainda investigou as especialidades dos médicos prescritores informadas nos processos. Observou-se que, entre as especialidades registradas, predominam os pneumologistas (33,8%). Este resultado corrobora as habilidades necessárias para o atendimento das condições clínicas mais frequentes nos pacientes com DPOC, a saber: DPOC de difícil controle, considerada quando o paciente apresenta duas ou mais internações, ou ainda idas à emergência causadas por exacerbação no último ano; paciente que faz o uso de corticoesteroide inalatório, beta-agonistas de longa duração ou broncodilatadores anticolinérgicos de longa duração; portadores da doença de forma muito grave, com volume expiratório forçado no primeiro segundo (VEF1) pós-uso de broncodilatador menor do que 30% do previsto; paciente que se apresenta sintomático, mesmo fazendo uso de terapia bem estabelecida.^19^

A especialidade em cirurgia torácica foi descrita em 5% das demandas. Esta especialidade é diretamente relacionada ao tratamento cirúrgico da DPOC, que prevê procedimentos como a bulectomia, a cirurgia redutora de volume pulmonar e o transplante pulmonar.^20^

Frequentemente, os medicamentos sofrem reajustes de valores, conforme regulação da ANVISA.^14^ O valor unitário pago pela SESA-PR para aquisição do brometo de tiotrópio aumentou 24% no decorrer do período considerado (2010 a 2016). A evolução dos gastos públicos com este medicamento é consideravelmente menor do que seu custo para aquisição individual (pelo próprio paciente). Como exemplo, no ano de 2016, a SESA-PR pagava aproximadamente R$ 179,00 pela unidade do brometo de tiotrópio, enquanto o paciente desembolsava, em média, R$ 293,00.

Tomando como base o salário mínimo de cada ano e o custo médio do brometo de tiotrópio, obteve-se média de valores do medicamento para compra pelo próprio paciente. É possível observar que o custo do medicamento corresponde a um valor alto da renda da população. Por exemplo, no ano de 2011, o brometo de tiotrópio representava 44,9% do salário mínimo vigente. Com este resultado, os gastos individuais com o medicamento impactam significativamente nas finanças dos pacientes assalariados. Ainda mais se considerarmos que estes são predominantemente idosos, aposentados e beneficiários do governo.

Enquanto o salário mínimo aumentou 57% no período considerado (2010 a 2016), o poder de compra decresceu quase 40% no mesmo período. Isso dificulta ainda mais a compra de forma individual, pelo próprio paciente.

Levando em consideração o Art. 196, da seção III da Constituição Federal, que diz que “a saúde é um direito de todos e um dever do Estado”, justifica-se o número crescente de demandas judiciais para o brometo de tiotrópio e seu atendimento.^21^

Nos Estados de São Paulo, Minas Gerais, Espírito Santo, Ceará e o Distrito Federal, o brometo de tiotrópio foi incorporado nos protocolos clínicos estaduais para o tratamento da DPOC. Beneficiam-se deste medicamento os pacientes que possuem alto de risco de exacerbação e sintomas persistentes, mesmo fazendo o uso de beta-agonistas de longa duração.^16^

Estudos internacionais recentes confirmam o custo-efetividade do brometo de tiotrópio administrado isoladamente ou em associação com o olodaterol (beta-agonista de longa duração). Houve, em ambos os casos, melhora da função pulmonar, da qualidade de vida, redução das exacerbações e aumento da sobrevida dos pacientes.^22 - 24^

No período de 2010 a 2016, o brometo de tiotrópio representou 1,7% dos gastos totais com demandas judiciais atendidas pelo Estado do Paraná. Apesar de ser o medicamento mais demandado judicialmente no Estado, não é o que mais impacta financeiramente. Entretanto, no campo individual, o medicamento gera impacto financeiro expressivo para os pacientes com DPOC, uma vez que grande parte não teria condições de arcar individualmente com o tratamento, necessitando da assistência do Estado. A desburocratização viabilizada pela incorporação do brometo de tiotrópio no SUS reduziria os custos no Paraná, e nos demais Estados, e permitiria aos pacientes um acesso mais digno e rápido ao tratamento confirmadamente efetivo.

## CONCLUSÃO

Com a nova atualização da *Global Initiative for Chronic Obstructive Lung Disease,* a utilização dos broncodilatadores anticolinérgicos de longa duração, como o brometo de tiotrópio, surge como proposta de primeira linha para a terapia da doença pulmonar obstrutiva crônica.

Conforme tentativa de inserção do brometo do tiotrópio no Sistema Único de Saúde, indeferida em 2013, observa-se que o custo de aquisição deste medicamento seria menor do que a compra pelo setor de demandas judiciais do Estado, representando economia significativa de recursos públicos.

Neste estudo, foi possível observar a necessidade da revisão do protocolo clínico da doença pulmonar obstrutiva crônica (ainda de 2013) e de inserir novas opções terapêuticas na Relação Nacional de Medicamentos Essenciais. Entre as quais, sugere-se a inserção do brometo de tiotrópio. A inclusão deste medicamento se justifica pelo número de demandas judiciais, bem como pelo impacto financeiro que o medicamento gera quando adquirido individualmente.
